# Regulatory mechanism of macrophage polarization based on Hippo pathway

**DOI:** 10.3389/fimmu.2023.1279591

**Published:** 2023-11-28

**Authors:** Yuanqing Liu, Yina An, Gebin Li, Shuaiyu Wang

**Affiliations:** ^1^ The Clinical Department, College of Veterinary Medicine, China Agricultural University, Beijing, China; ^2^ Center of Research and Innovation of Chinese Traditional Veterinary Medicine, China Agricultural University, Beijing, China

**Keywords:** macrophages, Hippo, tumor microenvironment, polarization, regulation

## Abstract

Macrophages are found to infiltrate and migrate in a large number of Tumor-associated macrophages (TMEs) and other macrophages in the microenvironment of tumors and related diseases, and undergo phenotypic changes in response to a variety of cytokines, mainly including the primary phenotype M2 and the anti-tumor phenotype M1. The Hippo signaling pathway affects the development of cancer and other diseases through various biological processes, such as inhibition of cell growth. In this review, we focus on immune cells within the microenvironment of tumors and other diseases, and the role of the Hippo pathway in tumors on macrophage polarization in the tumor microenvironment (TME) and other diseases.

## 1 Introduction

The Hippo signaling system is an exceptionally well-preserved cascade signaling mechanism seen in both drosophila and mammals., which inhibits cell growth, and engages in various biological processes to regulate key target genes, including cell proliferation, cell survival, cell differentiation, cell fate determination, and organ size and tissue homeostasis (1, 2). Abnormal signaling in the Hippo pathway affects the development of cancer and other diseases (3). It is significantly involved in regulating both cell proliferation and apoptosis during tumor formation (4). By preventing nuclear translocation of Yes-associated protein (YAP) and PDZ-binding motif (TAZ), it reduces cell proliferation and encourages apoptosis, and therefore the inactivation of Hippo pathway is associated with tumor formation.

Macrophages are a special type of immune cells and one of the essential phagocytic cells in human body, which play a significant part in removing pathogens and waste cells, etc. It is presumed that tissue-resident macrophages represent the initial cells transformed by tumor cells, leading to the formation of preneoplastic M2-like TAMs. Following that, monocyte-derived macrophages are attracted and undergo polarization into M2-like TAMs within the TME, a process necessary for the progression of metastatic and malignant tumors (5). In the context of the inflammatory response, a large number of circulating monocytes are mobilized to the site of tissue damage, where they subsequently undergo differentiation into pro-inflammatory classically activated macrophages, frequently termed M1 macrophages. This differentiation is triggered by a range of cytokines, including Interferon-γ (IFN-γ) (6). Upon resolution of the inflammatory trigger, various immune cells generate factors like pro-catabolic lipid mediators and Th2-type cytokines, which induce a transformation of macrophages into an anti-inflammatory phenotype, typically denoted as M2 macrophages (7). The function and phenotype of macrophages can change in different microenvironments, a phenomenon known as macrophage polarization. The active status of macrophages at a specific time is referred to as macrophage polarization, but because of the flexibility inherent in macrophages, their polarization state is adaptable and can be modified by integrating multiple signals originating from other cells, tissues, and pathogens (8). Depending on their function and phenotype, macrophage polarization can be categorized into two types: M1 and M2 (9–11).

In recent years, an expanding body of research has offered some light on the critical role of the Hippo pathway in the progression of tumors. These studies have unveiled the significance of this pathway in driving various aspects of tumor development, thereby deepening our understanding of cancer biology and potential therapeutic interventions. In this review, we will focus on the mechanisms and important roles of the Hippo pathway in regulating macrophage polarization of tumor-associated diseases. Moreover, we will provide a concise overview of the Hippo pathway’s functional implications in diseases that are not associated with tumors.

## 2 Hippo pathway overview

### 2.1 The core of the Hippo pathway lies in the kinase cascade reaction

**Figure 1:**
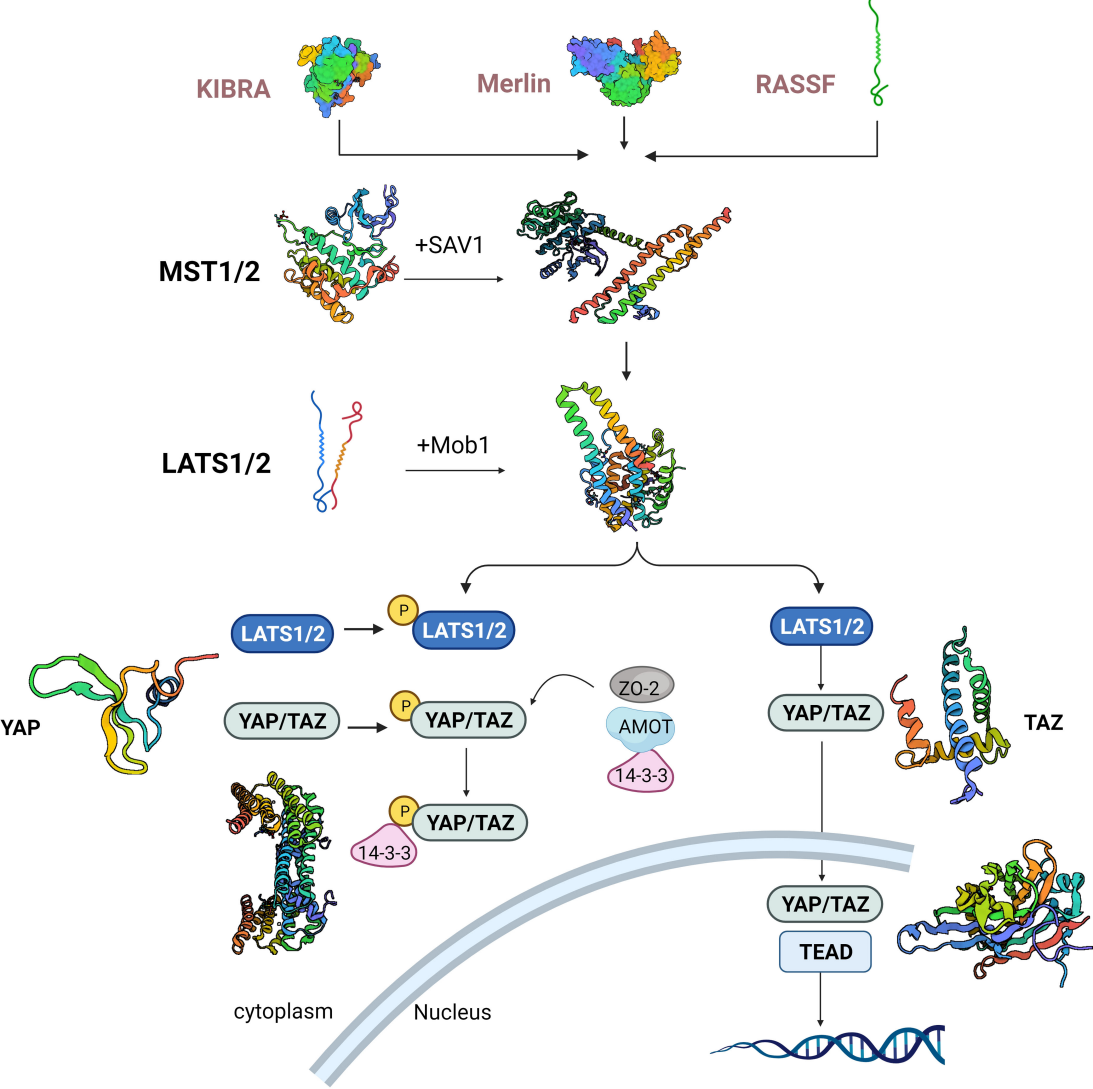
Hippo cascade activation signaling pathway. KIBRA, kidney and brain expressed protein; Merlin, moesin-ezrin-radixin-like protein. RASSF, ras-association domain family; MST1/2, Ste20-like kinases1/2; LATS1/2, large tumor suppressor 1/2; YAP, yes-associated protein; TAZ,PDZ-binding motif. SAV1, salvador family WW domain-containing protein.

The kinase cascade is at the center of the Hippo pathway, in which Ste20-like kinases1/2(MST1/2) kinase and Salvador family WW domain-containing protein (SAV1) form a complex that phosphorylates and activates large tumor suppressor 1/2(LATS1/2). The LATS1/2 kinase, in a subsequent step, phosphorylates and inhibits the transcriptional co-activators known as YAP and transcriptional coactivator with TAZ. Following dephosphorylation, YAP/TAZ undergo nuclear translocation (12). Hippo pathway activity can be subject to regulation at various levels: upstream molecules like Moesin-ezrin-radixin-like protein (Merlin), Kidney and brain expressed protein (KIBRA), Ras-association domain family (RASSF), and Ajuba, can influence MST1/2 expression and the phosphorylation of LATS1/2 (**Figure 1**). Additionally, 14-3-3, α-chain proteins, Angiomotin (AMOT), and Zona occludens 2 (ZO-2) participate in retaining YAP/TAZ within the cytoplasm through adhesion linkage or tight junctions. The phosphorylation and activity of MST1/2 and YAP/TAZ are under the control of phosphatases, while the stability of LATS1/2 and YAP/TAZ is managed through protein ubiquitination. Furthermore, the YAP/TAZ nuclear localization, acting as a downstream signal transducer within the Hippo pathway, can be influenced by substrate rigidity and cellular contractility (13, 14). Immune cells present within the TME have significant roles in the development of tumors. It is commonly recognized that these immune cells in the tumor vicinity can display either anti-tumor or pro-tumor roles. It is now clearly revealed by research that in addition to cancer cells, a repertoire of immune cells exist in TME, including stromal cells, endothelial cells and cancer-associated fibroblasts (15). In short, there are two types of immune cells in the tumor microenvironment: those that counteract tumor growth and those that facilitate it. The former group primarily comprises effector T cells including CD8+ cytotoxic T cells and effector CD4+ T cells, as well as natural killer cells, dendritic cells, M1-polarized macrophages, and N1-polarized neutrophils (16).

### 2.2 The role of Hippo pathway in tumor progression

**Figure 2:**
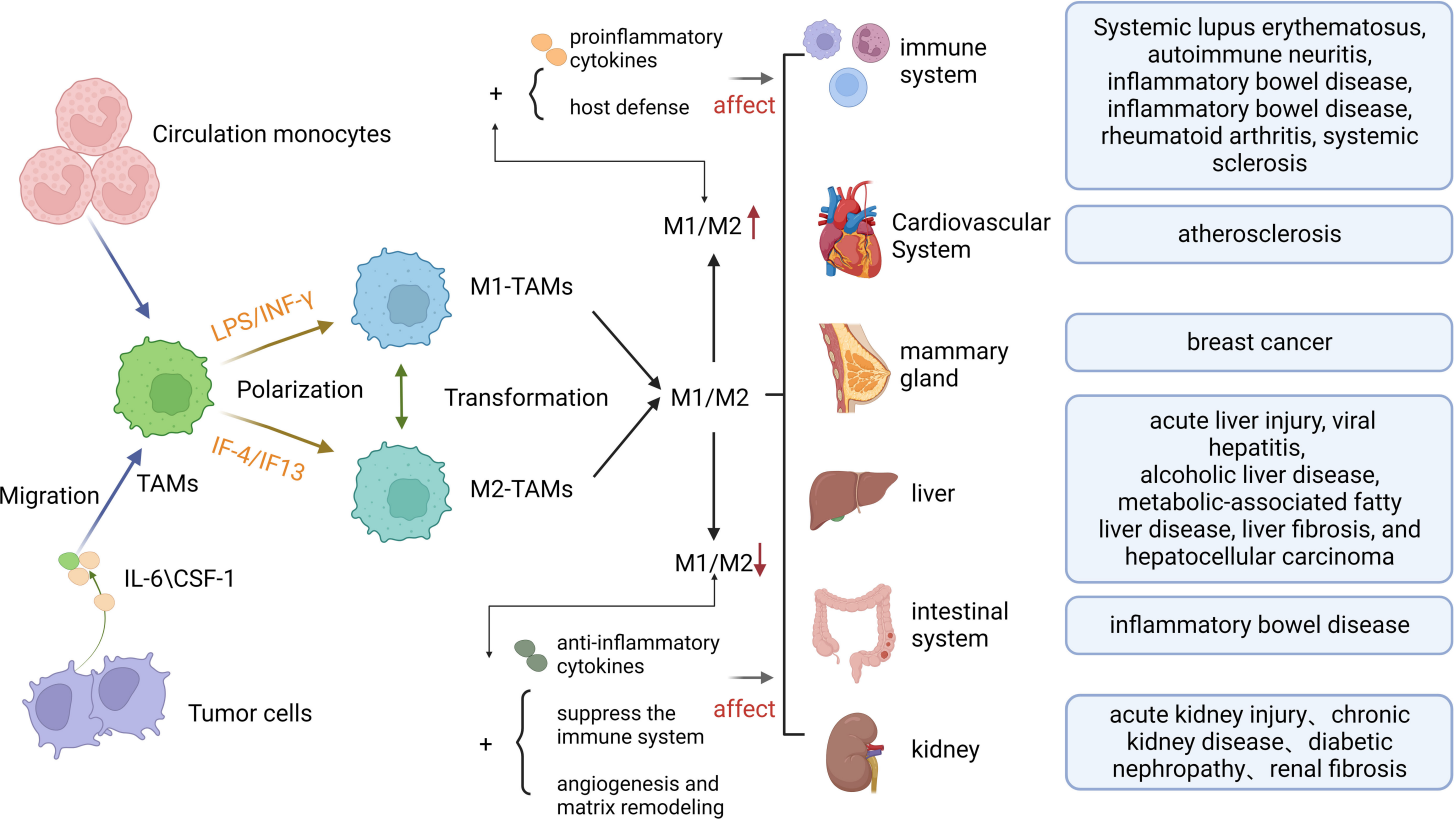
The Formation of Macrophage polarization and its Relationship with Human Diseases. TAMs, Tumor-associated macrophages; IL-6,Interleukin 6;CSF-1, macrophage colony-stimulating factor 1.

The Hippo pathway is also key to tumor progression. Studies have shown that inactivation of the Hippo pathway promotes the invasion and metastasis of cancer cells and engages in the formation of TME. The Hippo pathway also helps maintain tissue homeostasis and health by controlling cell growth and apoptosis. The malfunction of Hippo pathway can lead to loss of tissue homeostasis, thereby resulting in the development of tumors or other diseases. For relationship between macrophage polarization and human disease, see **Figure 2**. The Hippo pathway engages in regulating stem cell self-renewal and differentiation, and it plays a crucial role in maintaining tissue regeneration and repair capacity (17). The Hippo pathway also participates in the regulation of cell polarity, an asymmetry in the distribution of molecules within the cell. This process is vital for the control of cellular morphology and function, and has crucial implications for cell signaling, intercellular communication and tissue establishment (18). The Hippo pathway is a positive contributor to neuronal survival and axon formation in neurodegenerative diseases. The activation of the Hippo pathway can enhance neuronal survival and axon growth (19), reduce myocardial ischemia-reperfusion injury, inhibit myocardial fibrosis, and facilitate vascular development and stability (20). In a broader context, the Hippo pathway has established itself as a pivotal player in numerous diseases, marking it as a key focus for potential therapeutic interventions. Its multifaceted involvement across various medical conditions underscores its growing importance in the field of disease treatment and underscores the need for continued research to explore its therapeutic potential.

## 3 Hippo pathway and TAMs polarization

### 3.1 The origin of TAMs

Tumor-associated macrophages, often referred to as TAMs, are the predominant immune cells population that infiltrate and reside within the TME. They significantly dominate the immune cell composition within the TME (21–23). TAMs serve as the frontier for the phagocytosis of pathogens by tumor macrophages to initiate innate immunity and the products of TME to mediate tumor immunity. Immune suppression is the consequence of a compromised immune response orchestrated by TAMs (24). Within the TME, macrophages derived from circulating monocytes are a significant subset of immune cells. These macrophages exhibit distinct polarization states, with classically activated macrophages (M1) serving as “beneficial” macrophages. M1 macrophages are known for generating pro-inflammatory cytokines and reactive oxygen/nitrogen species, which are essential for host defense and the efficient elimination of tumor cells. In contrast, M2-polarized macrophages, while still a part of the TME, exhibit a different profile. They produce anti-inflammatory cytokines and actively suppress the immune system’s surveillance against tumor cells. Additionally, M2 macrophages promote processes like angiogenesis and matrix remodeling, thereby facilitating tumor progression and metastasis (25). In essence, the polarization of macrophages within the TME plays a pivotal role in influencing the tumor microenvironment’s dynamics and can either support host defense or contribute to tumor growth and metastasis. Since tumor behaviors interact with the TME to support tumor growth and immune evasion, they should be analyzed in the context of their microenvironment (26). Within the TME, TAMs are regarded as among the most crucial immune cells, and their role in providing innate as well as adaptive immunity to the host is of significant importance (27). As a prominent presence among immune cells within the tumor microenvironment (TME), macrophages exhibit both phagocytic capabilities and serve as vital antigen-presenting cells. Their substantial involvement in the inflammatory cascade is driven by their ability to release pro-inflammatory cytokines when faced with pathogens or tissue damage, making them integral components for preserving overall homeostasis (28, 29). Macrophages can promote tumor development, enhance angiogenesis and stimulate tumor migration through a variety of mechanisms (30, 31). Therefore, the behavioral changes of macrophages in the TME deserve further research.

### 3.2 Hippo pathway regulates TAMs polarization

Macrophage polarization, at any given moment, signifies the activation state of a macrophage, yet owing to the adaptability of macrophages, their polarization status can be modified through the integration of various factors (8). Research has revealed that the polarization of macrophages is governed by multiple molecular mechanisms, mainly including Toll-likereceptor4 (TLR4)/Nuclear factor kappa-B (NF-κB), JAK/STATs, TGF-β/Smads, peroxisome proliferator-activated receptor gamma (PPARγ), Notch, Hippo and miRNA signaling pathways (32). Signal transduction pathways alter the disease microenvironment through mechanisms of activation or inactivation, thereby indirectly altering the macrophage phenotype to influence disease progression. The YAP from the Hippo signaling cascade, together with TAZ of transcriptional coactivator can respond to hard substrate signaling. To illustrate, when subjected to signaling from a rigid substrate, YAP and TAZ activation leads to their nuclear translocation. Consequently, this regulatory process orchestrates the activation of genes associated with glucose and amino acid metabolism, which play a critical role in determining the polarization state of macrophages. This metabolic adaptation is essential for their functional specialization within various immune responses. These molecular changes induce shifts in macrophage metabolism to better suit the microenvironment, creating acidic and inflammatory conditions that influence macrophage polarization and effector functions, including acidification and inflammatory microenvironments that can direct macrophage polarization and effector function, alter macrophage metabolism to adapt to the microenvironment (33). Since abnormal signaling of the Hippo pathway affects the development of tumors and other diseases by influencing the M1/M2 polarization status of macrophages, as well as the overall prognosis, it is significant to investigate the roles of the Hippo pathway on macrophage polarization mechanisms. Macrophages exhibit a remarkable ability to adapt and undergo coordinated changes in gene expression when exposed to signals from the TME. This high plasticity endows them with unique native phenotypes that possess anti-inflammatory and immunosuppressive capabilities (34). Consequently, TAMs wield a significant influence over the initiation of tumors and contribute significantly to the complex landscape of tumorigenesis. M2 macrophage polarization further suppresses immunity by inhibiting T-cell proliferation and associated cytokine production (35, 36). Tumors have been reported to acquire an M2-like phenotype if TAM acts through T cells, cancer cells, or other interacting cell types within the TME (37, 38). The TME is primarily characterized by the prevalence of M2-like TAMs. However, enhancing the activity of M1-like TAMs can locally inhibit tumor growth. This phenotypic shift in macrophages is influenced by various factors, including cytokines and metabolites produced by tumor cells. For instance, lactate, a metabolic byproduct of tumor cells, drives macrophage polarization toward the M2 phenotype via Hypoxia-inducible factor-1α (39). Therefore, there is significant potential in modifying the equilibrium between M1-like and M2-like TAMs within the TME as a promising strategy for TAM-focused cancer immunotherapy (40). This approach holds the key to reshaping the immune landscape in the tumor microenvironment and, in turn, impacting tumor progression.

The mediators of Hippo signaling, MST1/2 and YAP/TAZ, have a significant impact on in modulating the phenotype of macrophages, thereby exerting a significant influence on cancer prognosis. YAP/TAZ, a key factor of the Hippo pathway, regulates genes in macrophages, thereby altering the phenotype of macrophages and influencing macrophage migration. In the context of hepatocellular carcinoma, YAP has been identified as a factor that stimulates the migration of macrophages, both in *in vivo* and *in vitro* (41). Additionally, Lee and his research team have uncovered the regulatory role of YAP/TAZ in approximately 66 genes associated with various functions, including differentiation, immunity, cell development, and metabolism. Some of these genes include Myoblast determination protein (MyoD), Lymphocyte function-associated antigen 1 (LF-A1), Peroxisome proliferator-activated receptor gamma (PPARγ), The finger of the cerebellum 1 (Zic1), along with roughly 69 other genes that influence macrophage behavior (42). Similarly, the products of M2 macrophages can also influence disease progression via the Hippo pathway. In their study, Yuan et al. presented evidence that exosomal miR-31-5p originating from M2 macrophages can either promote or hinder the progression of Oral Squamous Cell Carcinoma (OSCC) by influencing the tumor suppressor gene LATS2. This regulatory effect occurs through the modulation of the Hippo signaling pathway. These findings offer promising prospects for the development of new molecular therapy targets in the context of OSCC (43), potentially opening up avenues for innovative treatment approaches.

**Figure 3:**
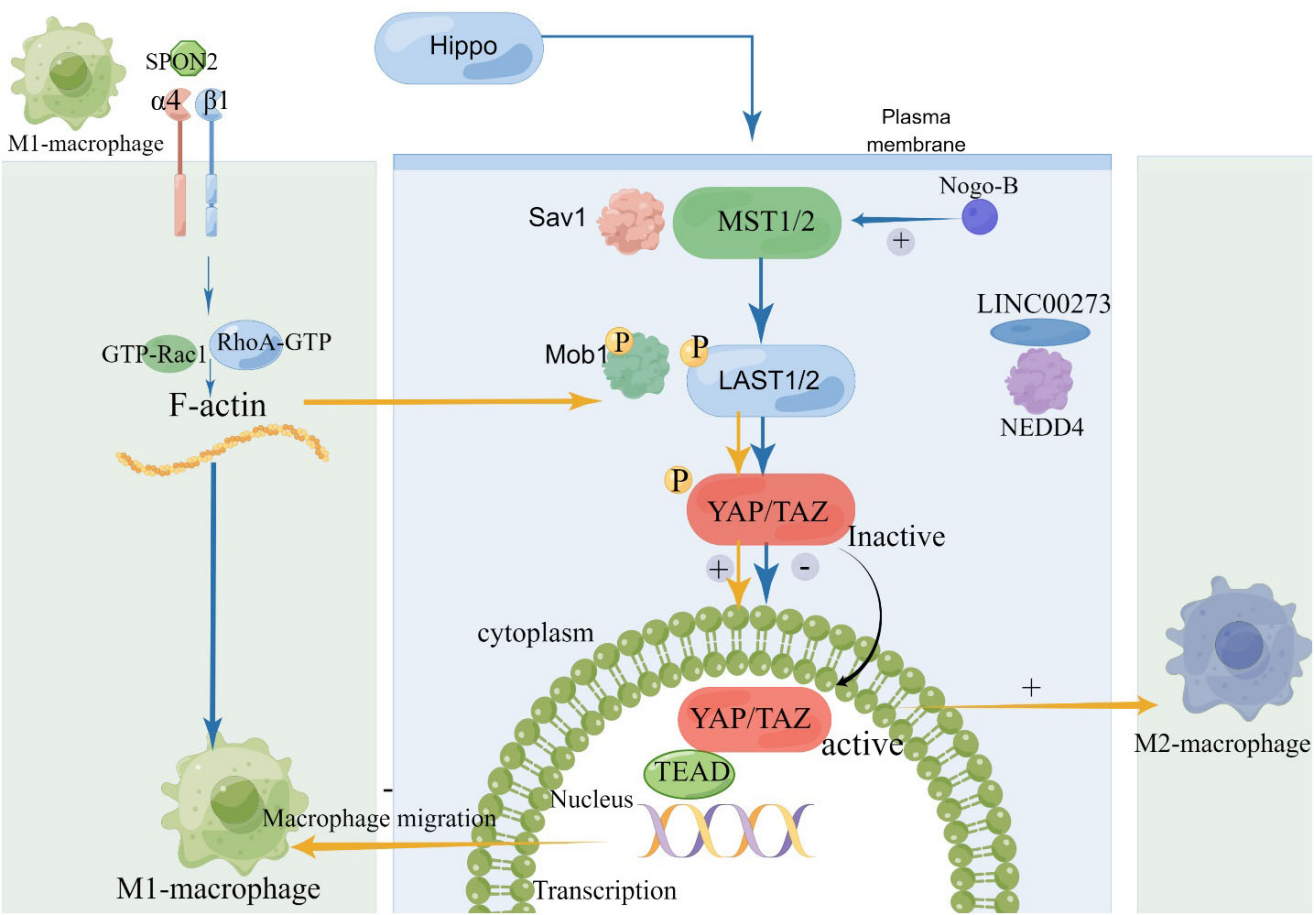
The Relationship between Hippo Pathway and Macrophage polarization. SPON2, Spondin2; NEDD4, neuronally expressed developmentally downregulated 4.

Different chemokines and tissue structures can alter the M1/M2 polarization state by binding to key factors of the Hippo pathway, see **Figure 3**. Totaro et al. made a noteworthy observation that the Hippo signaling pathway, which involves mediators MST1/2 and YAP/TAZ, is susceptible to regulation by various environmental and biological cues. These cues encompass alterations in nutrient levels and changes in cell polarity (44). In a separate study, Zhang et al. proposed an intriguing connection between the extracellular matrix secretory protein Spondin2 (SPON2) and the recruitment of M1 TAMs. Their research indicated that SPON2 interacts with integrin α4β1 to promote the recruitment of M1 TAMs. Interestingly, the introduction of recombinant Spondin2 (rSPON2) protein led to a significant increase in the translocation of YAP into the nucleus within human monocyte (THP-1) cells, coinciding with the induction of the M1 phenotypic polarization (41). These findings underscore the complex interplay between the Hippo pathway and other cellular processes, shedding light on potential therapeutic targets. *In vitro* studies, Nogo-B and TMEs Tumor-associated macrophage can effectively reduce M1 Macrophage polarization. Macropechanistic investigations have indicated that Nogo-B forms a binding relationship with MST1/2, which subsequently results in an elevation in MST1/2, LAST1, and YAP phosphorylation levels. This cascade of events culminates in a reduction in YAP activity. Rao J put forth a hypothesis that posits the absence of Nogo-B results in a limitation of phosphorylation events involving kinases MST1/2 and LATS1. This, in turn, facilitates the nuclear translocation of YAP and subsequently inhibits the process of M1 polarization (45). LINC00273 recruits neuronally expressed developmentally downregulated 4 (NEDD4) to promote LATS2 ubiquitination and degradation, and activates YAP, thereby inactivating the Hippo pathway, and M2-polarized macrophages promote lung adenocarcinoma (LUAD) cell invasion and migration and tumor metastasis *in vivo* (46). A Rum Kim found that TAZ stimulated macrophage infiltration, leading to IL-6 production and promoting M1 macrophage polarization, thereby inducing liver regeneration (47, 48). To manage pulmonary inflammation in the context of mechanical ventilation (MV), it’s crucial to maintain a balance between macrophage M1 and M2 polarization. Qiong Luo’s report marked the first time that YAP induction in macrophages played a role in pulmonary inflammation during MV, primarily by influencing the M1/M2 polarization. Kidney fibrosis is known to be caused by M2 macrophage polarization. Their findings showed that Wnt5a induced kidney fibrosis by triggering macrophage M2 polarization mediated by YAP/TAZ. Liu et al. demonstrated that ubiquitin-specific peptidase 10 (USP10) inhibited YAP1 ubiquitination and degradation to promote cysteine rich angiogenic inducer 61 (Cyr61) expression, which induced immune escape and promoted the growth and metastasis of pancreatic adenocarcinoma (PAAD).

Deletion of key factors of the Hippo signaling pathway leads to decreased expression of cytokines or genes, which in turn affects macrophage polarization status and alters cancer progression. Wantae Kim’s findings revealed that Hippo-deficient hepatocytes have actively influenced their immediate microenvironment. They not only triggered the infiltration of macrophages but also orchestrated their activation, resulting in a diverse spectrum of M1 and M2 phenotypes. Notably, these orchestrated activities were found to be critically reliant on the presence of Monocyte Chemoattractant Protein 1 (MCP1). YAP expression has demonstrated associations with both cell-autonomous and non-autonomous mechanisms in M2 TAM polarization and tumorigenesis (48). In colon cancer cells, when YAP expression is downregulated, it leads to inhibition of tumor formation, metastasis, and the development of cancer stem cell-like cells. Simultaneously, this downregulation is accompanied by reduced levels of cytokines like IL-4 and IL-13, which are important inducers of M2 polarization production. Anakk found that bile acids activated YAP through a scaffold protein IQGAP1-dependent pathway, resulting in enhanced IQ motif-containing GTPase-activating protein 1(IQGAP1) and nuclear YAP expression and promoting liver growth and hepatocarcinogenesis (49). In summary, it is reported that the role of the YAP signaling pathway in regulating the polarization of tumor-associated macrophages (TAMs). It appears that when YAP is silenced in THP-1 cells, there is a significant reduction in M2 markers such as ARG1 and CD23, as well as other factors related to M2 polarization like β-catenin, ATK, and NF-κB. This suggests that YAP promotes the polarization of TAMs towards the M2 phenotype, which is associated with a tumor-supportive environment. It also mentions that YAP activation in hepatocytes can lead to the formation of tumor-initiating cells and the polarization of peripheral TAMs towards the M2 phenotype (50). On the other hand, in pancreatic ductal adenocarcinoma (PDAC), deleting YAP seems to promote TAMs polarization to the M1 phenotype, characterized by increased expression of inducible NOS (iNOS) but not arginase (51). Overall, these studies highlight the importance of the MST1/2-Last1/2-YAP pathway in mediating the crosstalk between tumor cells and TAMs, ultimately influencing the polarization of TAMs towards either the M1 or M2 phenotype, which can have significant implications for tumor progression.

### 3.3 Exosomes alter the polarization of macrophages by Hippo pathway

In addition, TAMs reprogram macrophages by carrying exosomes from tumor cells, thereby creating a microenvironment for tumor growth. In summary, M2 macrophages have a substantial impact on altering cancer cells. They achieve this by releasing exosomes, which serve as vehicles for promoting tumor growth and facilitating metastasis, ultimately contributing to the progression of cancer. Exosomes derived from M2 macrophages specifically promote the advancement of colon cancer and gastric cancer (52, 53). Moreover, secreted exosomes carrying miR-21-5p can be conveyed from mesenchymal stem cells (MSCs) to cancer cells and macrophages, driving macrophage M2 polarization and triggering tumor cell proliferation while inducing immunosuppression within the TME. These findings underscore the significant role of M2 macrophages and exosomes in tumor progression and immune regulation (54). The transcription factors STAT3/6, Krueppel-like factor 2/4, and IRF3/4 play a part in driving the activation of M2-like tumor-associated macrophages (TAMs). They are instrumental in promoting ARG1-dependent arginine metabolism, which is a characteristic feature of these TAMs (52). Additionally, miR-511-3p, miR-155-5p and miR-142-3p have all been shown to limit the activation of m2-like macrophages in tumors (55–57). Current studies suggest that exosomes mediate the polarization of macrophages through the Hippo pathway. The phenomenon of phenotypic changes in macrophage polarization has been found to occur in other diseases besides tumors. To explore the potential mechanism by which MSC-derived exosomes inhibit M1 polarization in macrophages, performed target gene prediction for different expressions of tRFs, followed by KEGG pathway analysis. KEGG analysis showed that these target genes were mainly concentrated in inflammation-related pathways, including Hippo, MAPK and TGF-β signaling pathways (58). Notably, found that the lung adenocarcinoma cell exosome miR-19b-3p is regulated by YAP, the downstream core protein of LATS2 in the Hippo pathway. Currently, we have found that mir-19b-3p induced exosomal release from M2 macrophages promotes LATS2 ubiquitination and activation of YAP in lung adenocellular cancer cells (46). These findings provide a new possibility to further explore the crosstalk relationship between Hippo pathway and exosomes. Extracellular vesicles (ev) serve as essential mediators for intercellular communication among macrophages and various cell types. Through their actions, they instigate significant phenotypic alterations in the recipient cells, illustrating the remarkable role of EVs as messengers in cellular signaling pathways (59, 60). Furthermore, Hutcheson et al. suggested that macrophages promoted calcification remodeling not only indirectly, but also directly by releasing calcified ev (61).

## 4 Hippo pathway and macrophage polarization associated with non-neoplastic diseases

MST1/2 serves as the upstream regulator of YAP/TAZ in the Hippo signaling pathway, and it has been demonstrated to influence macrophage phenotype regulation. Recent investigations have underscored the importance of YAP/TAZ signaling in molding the functionality of macrophages, influencing their polarization and overall phenotype. Nevertheless, the precise mechanism governing these changes remains enigmatic due to the multitude of upstream signals that have the potential to regulate YAP/TAZ expression and activity. Unraveling this intricate web of interactions represents a vital area of ongoing research in understanding macrophage biology. Further research is required to elucidate the intricate details of this regulatory network within macrophages (44). It was found that the key factors of the Hippo pathway, MST1/2 and YAP/TAZ, not only regulate macrophage polarization status in tumor-associated diseases, but also significantly influence macrophage polarization in non-tumor-associated diseases. The absence of YAP leads to a reprogramming of macrophage phenotypes and has a substantial impact on the regulation of M1/M2 macrophage polarization. This reprogramming, in turn, contributes to enhanced recovery in conditions such as infarct healing, lung injury, and inflammatory bowel disease (IBD), signifying the pivotal role of YAP deficiency in these therapeutic contexts.

Furthermore, in macrophages with a proinflammatory phenotype, there was an upregulation in the expression of YAP/TAZ. And the activation of YAP enhanced the proinflammatory response, whereas the genetic deletion of YAP and TAZ suppressed the proinflammatory response and enhanced the repair response. Within the context of myocardial infarction, the shift in macrophage polarization brings about a notable reduction in cardiac fibrosis and hypertrophy, ultimately resulting in enhanced cardiac function (62). In the macrophage population, the absence of MST1/2 exacerbates cardiac dysfunction following a myocardial infarction. Specifically, Mice with the absence of MST1/2 in their macrophages display significant increases in left ventricular end-diastolic and end-systolic volumes, coupled with a notable decline in ejection fraction (63). This highlights the pivotal role of MST1/2 in modulating post-infarction cardiac outcomes. Specifically, post-infarction repair was impaired in mice with MST1/2-specific efficiency in macrophages compared to wild-type mice (64). Furthermore, the conversion of M2-like TAMs to the M1-like phenotype holds promise for enhancing T lymphocyte infiltration into tumors, thus amplifying their effectiveness in eradicating cancer cells. However, it’s important to note that an overly aggressive activation of M1-like TAMs can lead to chronic inflammation, which in turn may contribute to the development of conditions like atherosclerosis and other chronic inflammatory diseases. Nonetheless, the controlled transformation of M2-like TAMs into M1-like TAMs, under specific conditions, has the potential to trigger tumor regression, offering a valuable avenue for targeted cancer therapy (40). This delicate balance underscores the complexity of TAMs’ role in cancer and inflammation.

In the context of IBD, research involving YAP-deficient mice has revealed an increased presence of pre-resolution polarized macrophages within their colonic tissue. This observation highlights the impact of YAP deficiency on the macrophage population in the context of IBD. Interestingly, this increased presence of macrophages with a specific polarization profile, which appears to offer protection against IBD. In human IBD patients, colonic samples also show a significant presence of macrophages, highlighting their role in both triggering and resolving inflammation associated with the disease (65). Recent research has provided insight into the involvement of YAP/TAZ and MST1/2 in macrophages and dendritic cells (DC) within the context of intestinal inflammation, including situations like intestinal infections and IBD. YAP, in particular, contributes to preserving the balance of pro-inflammatory (PIM) and pro-resolving (PRM) macrophages. Notably, YAP deficiency has been found to alleviate colitis induced by dextran sodium sulfate (DSS), indicating its potential as a therapeutic target (66). In essence, these findings underscore the intricate involvement of YAP/TAZ and MST1/2 in regulating macrophage behavior in the intestinal inflammatory setting (67). Targeting these pathways may hold promise in the development of treatments for conditions like IBD, by modulating macrophage polarization and inflammation levels. Pharmacological inhibition of YAP/TAZ suppresses the expression of pro-inflammatory genes (IL-1β and IL-12β) (24). Pro-inflammatory cytokine concentrations of IL-6, TNF-α and IL-1β were elevated in cell supernatants after LPS/IFN-γ induction. Levels of anti-inflammatory cytokines IL-10, IL-12 and IL-13 associated with M2 were remarkably reduced after LPS/IFN-γ induction (68).

Recent research have shown YAP plays a pivotal role as a regulon of macrophage function in the pathogenesis of liver disease. Notably, the selective inhibition of YAP in macrophages through Dopamine receptor D2 (DRD2) antagonism has demonstrated promise in preventing liver fibrosis, making it a potential candidate for treating nonalcoholic steatohepatitis (NASH) (69). Additionally, Kupffer cells (KCs) have been identified as contributors to the development of NASH through the enhancement of pro-inflammatory cytokine production (70). YAP is crucial in governing liver inflammation triggered by macrophage Nogo-β. Nogo-B fosters innate inflammation associated with macrophages and plays a role in ischemia-reperfusion-induced liver injury by activating the MST-mediated Hippo/YAP pathway. This pathway presents a potential treatment target for the clinical management of hepatic ischemia-reperfusion injury (IRI). Moreover, innate Toll-like receptor 4 (TLR4) is a key player in the inflammation induced by insulin resistance (IR) in the liver, linking it to the broader spectrum of liver inflammation (71, 72). When primary mouse macrophages are exposed to TLR4 ligands like LPS or HMGB-1, interfering with Nogo-B has several notable effects. It leads to a reduction in the production of Reactive Oxygen Species (ROS), which are molecules involved in various cellular processes, including inflammation. Furthermore, this disruption of Nogo-B also dampens the inflammatory response mediated by TLR4 and impedes the polarization of macrophages toward the M1 phenotype. In simpler terms, targeting Nogo-B in these macrophages appears to mitigate oxidative stress, curb excessive inflammation triggered by TLR4, and hinder the development of pro-inflammatory M1 macrophages.

### 4.1 MSCs cells altering the polarization state of macrophages via the Hippo pathway

MSCs cells may alter the macrophage polarization state via the Hippo pathway. Mesenchymal stem cells (MSCs) play a key part in the activation of the macrophage Hippo pathway. This activation, exerts control over NLRP3 activation by establishing direct interactions between YAP and β-linked proteins. It also oversees XBP1-mediated NLRP3 activation, resulting in the reconfiguration of macrophage activation towards an anti-inflammatory M2 phenotype. This process underscores the pivotal role of MSCs in modulating macrophage behavior and promoting an anti-inflammatory response. Disruption of myeloid YAP or β-linked proteins in MSC-transfected mice exacerbates IR-triggered liver inflammation, enhances NLRP3/caspase-1 activity, and reduces the M2 macrophage phenotype. NLRP3 is critical for balancing M1/M2 macrophage polarization in YAP-β-cyclin-mediated regulation (73). In their study, Huang et al. uncovered that YAP plays a significant role in inducing M2-like TAMs polarization within colorectal cancer. This polarization, enhanced TAMs’ tumor-initiating capabilities. Importantly, they also demonstrated that when YAP was blocked in combination with 5-fluorouracil, it led to a reduction in tumorigenesis and prevented both TAM polarization and TAM-related treatment resistance (74). Furthermore, exposure to arsenite was found to elevate miR-15b levels, promoting M2 polarization in THP-1 cells. Through the transfer of high levels of miR-15b from arsenite-exposed THP-1 (As-THP-1) cells to hepatocellular carcinoma (HCC) cells via miR-15b-containing extracellular vesicles (EVs), the Hippo pathway’s activation was inhibited by targeting LAST1. This inhibition, in turn, facilitated HCC cell proliferation, migration, and invasion (75). These findings underscore the intricate interplay between YAP, miR-15b, and the Hippo pathway in the context of cancer progression. In summary, the macrophage polarization status in non-tumor-related diseases can be indirectly altered by the activity status of MST1/2 and YAP/TAZ, two key regulators of the Hippo pathway.

## 5 Conclusions and future development

M1/M2 polarization of macrophages serve as a key factor in the TME and other related diseases. To be specific, M1 macrophages exert an antimicrobial phagocytic effect and M2 macrophages affect tumor and other diseases by suppressing inflammation, repairing tissue, and tissue structural reconstruction. However, Kim.W et al. found that the genetic deletion of Mst1 and Mst2 in hepatocytes (DKO) resulted in the onset of HCC. This condition was associated with a substantial increase in Mcp1 expression and the extensive infiltration of macrophages displaying a combination of both M1 and M2 phenotypes. These findings cast light upon the complex dynamics of macrophage involvement in HCC development when MST1 and MST2 are deficient in hepatocytes. The presence of mixed M1/M2 type macrophages in the disease, and its role in tumors and diseases remain unclear (29). Therefore, continued research is required to investigate the role of mixed M1/M2 type macrophages in the disease. This review summarizes how changes in the activity of key factors MST1/2 and YAP/TAZ in the Hippo pathway lead to polarization of TAMs. Meanwhile, different chemokines and tissue structures can alter M1/M2 polarization status by binding to the key factor of MAT1/2 with YAP/TAZ of the Hippo pathway. In addition, the Hippo pathway is also closely associated with macrophage polarization in non-tumor related diseases, such as infarct healing, lung injury or IBD.

Based on the above findings, it is known that the Hippo pathway not only recruits TAMs to tumors and their adjacent tissues through YAP-mediated recruitment, but also directly regulates macrophage polarization. Hence, targeting YAP emerges as a promising strategy for precision cancer therapy. This approach holds the potential to offer more targeted and effective treatments for various types of tumors by disrupting YAP-related pathways (10). Although YAP/TAZ is widely and commonly activated in tumors and other diseases, and plays a key role in tumor growth regulation, it has great potential as a therapeutic target. However, due to technological limitations and YAP/TAZ’s inherent ability to regulate multiple stem cell activities and tissue regeneration, direct targeting of YAP/TAZ for tumor treatment is facing major strategic challenges. Additionally, indirect targeting of YAP/TAZ aims to target the signaling pathways that abnormally regulate YAP/TAZ activation in tumor cells, which theoretically has less impact on the functional requirements of YAP/TAZ by damaging normal cells, and therefore has better precision in terms of strategy. In contrast, the available targets and the basis of target selection for indirect targeting of YAP/TAZ remain unclear, mainly because the specific mechanism of abnormal regulation of YAP/TAZ in tumors remains unknown. However, since the specific mechanism of the aberrant regulation of YAP/TAZ in tumors is far from clear, there are few targeting experiments for YAP/TAZ, MST1/2 and TAZ. The specific mechanism of macrophage polarization by key factors at all levels of the Hippo pathway is not yet perfectly defined. In our future studies, we will further investigate and refine the specific mechanisms of abnormal regulation of YAP/TAZ in tumors, and focus on the specific mechanisms of polarization of TAMs by key factors at all levels of the Hippo pathway, so as to optimize the mechanisms of immune regulation, dig deep into the molecular mechanisms and biological properties of tumor-associated macrophages, better understand their functions and regulatory mechanisms and provide references for the development of more effective therapeutic approaches.

In recent years, the diverse functions of macrophages have attracted a great deal of attention from researchers. Macrophages facilitate tumor growth and metastasis, identify and destroy tumor cells and engage in many biological processes such as healing and regeneration. Therefore, future studies should provide more insights into the specific role of macrophages in these processes. Secondly, the Hippo pathway or YAP and TAZ-targeted therapies may be conducive to the treatment of pancreatic cancer, atherosclerosis and other diseases. However, the efficacy and patient prognosis of targeting the Hippo pathway is not significant due to the complex process of tumorigenesis and the interactions between the Hippo pathway and tumor and other disease mechanisms, which have not been elucidated. Therefore, it is necessary to investigate in depth the mechanism of the Hippo pathway MST1/2 and YAP/TAZ on macrophage polarization so as to better test the efficacy of targeting the Hippo pathway in clinical practice and further improve the accuracy of targeted therapy. In tumor cells, aberrant Hippo signaling pathways can influence the infiltration and activation of immune cells by affecting their microenvironment, thereby affecting the body’s anti-tumor immune response. Therefore, the impact on the body’s immune system needs to be taken into account when targeting tumors.

In conclusion, the function of the non-classical Hippo pathway in the immune system requires further exploration. A profound understanding of the important role of non-classical Hippo signaling pathways in the preservation of immune system homeostasis will provide drug targets and therapeutic strategies for the treatment of infections, autoimmune diseases, ageing and tumors. Therefore, future research should focus on the diverse functions of macrophages and the mechanisms of non-classical Hippo pathways in the immune system for further advance the development of disease therapy.

## Author contributions

YL: Visualization, Writing – original draft, Writing – review & editing. SW: Funding acquisition, Writing – review & editing. YA: Writing – review & editing. GL: Writing – review & editing.

## Glossary

**Table:**

(TAMs)	tumor-associated macrophages
(TAM)	the tumor microenvironment
(IFN-γ)	interferon-γ
(MST1/2)	ste20-like kinases1/2
(SAV1)	salvador family WW domain-containing protein
(LATS1/2)	large tumor suppresspr 1/2
(YAP)	yes-sociated protein
(TAZ)	transcriptional coactivator with PDZ-binding motif
(Merlin)	moesin-ezrin-radixin-like protein
(KIBRA)	kidney and brain expressed protein
(RASSF)	ras-association domain family
(AMOT)	angiomotin
(ZO-2)	zona occludens 2
(NK)	natural killer cells
(DCs)	dendritic cells
(TLR4)	toll-likereceptor4
(NF-κB)	nuclear factor kappa-B
(PPARγ)	peroxisome proliferator-activated receptor gamma
(VEGF)	vascular endothelial growth factor
(HIF-1)	hypoxia inducible factor-1
(MyoD)	myoblast determination protein
(LF-A1)	lymphocyte function-associated antigen 1
(Zic1)	the finger of the cerebellum 1
(OSCC)	oral squamous cell carcinoma
(SPON2)	spondin2
(rSPON2)	recombinant Spondin2
(NEDD4)	Neuronally expressed developmentally downregulated 4
(THP-1)	human monocyte
(LUAD)	lung adenocarcinoma
(MV)	mechanical ventilation
(USP10)	ubiquitin-specific peptidase 10
(Cyr61)	cysteine rich angiogenic inducer 61
(MCP-1)	monocyte chemoattractant protein-1
(IQGAP1)	IQ motif-containing GTPase-activating protein 1
(ARG1)	Arginase1
(TICs)	Tumor-initiating cells
(PDAC)	Pancreatic ductal adenocarcinoma
(iNOS)	inducible NOS
(IBD)	inflammatory bowel disease
(DRD2)	Dopamine receptor D2
(NASH)	nonalcoholic steatohepatitis
